# Direct and Propagated Effects of Small Molecules on Protein–Protein Interaction Networks

**DOI:** 10.3389/fbioe.2015.00119

**Published:** 2015-08-24

**Authors:** Laura C. Cesa, Anna K. Mapp, Jason E. Gestwicki

**Affiliations:** ^1^Program in Chemical Biology, Life Sciences Institute, University of Michigan, Ann Arbor, MI, USA; ^2^Department of Chemistry, University of Michigan, Ann Arbor, MI, USA; ^3^Department of Pharmaceutical Chemistry, Institute for Neurodegenerative Diseases, University of California San Francisco, San Francisco, CA, USA

**Keywords:** multi-protein complexes, allostery, PPI inhibition

## Abstract

Networks of protein–protein interactions (PPIs) link all aspects of cellular biology. Dysfunction in the assembly or dynamics of PPI networks is a hallmark of human disease, and as such, there is growing interest in the discovery of small molecules that either promote or inhibit PPIs. PPIs were once considered undruggable because of their relatively large buried surface areas and difficult topologies. Despite these challenges, recent advances in chemical screening methodologies, combined with improvements in structural and computational biology have made some of these targets more tractable. In this review, we highlight developments that have opened the door to potent chemical modulators. We focus on how allostery is being used to produce surprisingly robust changes in PPIs, even for the most challenging targets. We also discuss how interfering with one PPI can propagate changes through the broader web of interactions. Through this analysis, it is becoming clear that a combination of direct and propagated effects on PPI networks is ultimately how small molecules re-shape biology.

## Introduction

**Figure 1:**
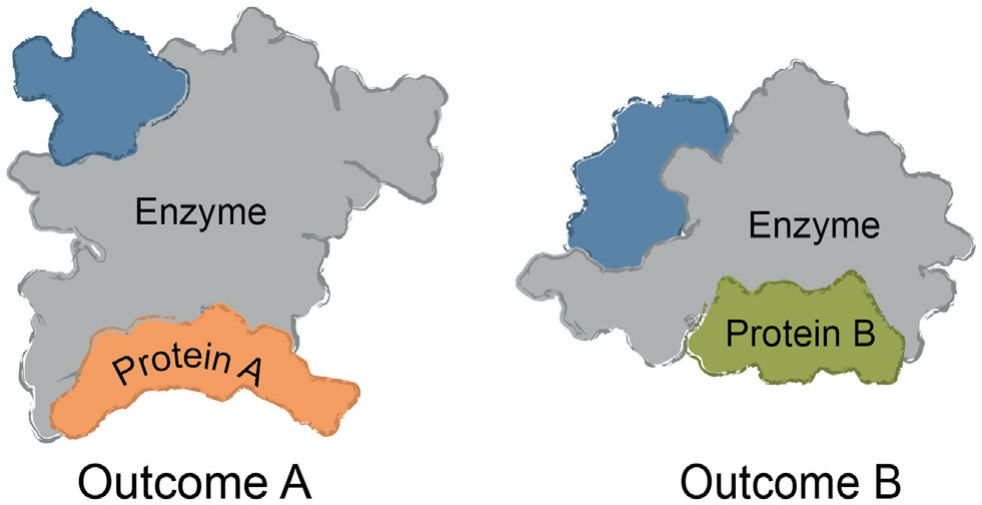
**Common themes in multi-protein complex assembly**. Multi-protein complexes are typically assembled around a core enzyme, while interactions with adaptor, scaffolding, and other partner proteins affect the overall function of the complex. Binding to ligands or macromolecules can trap one conformer of the complex. For example, binding to Protein A might alter the conformation of the enzyme to impact turnover or selectivity. Likewise, binding to Protein B (rather than Protein A) might be favored by a small molecule, changing the function and composition of the complex.

Multi-protein complexes are often assembled around a central enzyme, such as a kinase, phosphatase, protease, or nuclease. Interactions of the “core” enzyme with adaptor and scaffolding proteins often direct it to specific subcellular locations and/or regulating its enzymatic activity (Höhfeld et al., 2001; Young et al., 2009; Romero et al., 2011). For instance, protein A might trap a given conformer of the enzyme to favor a specific outcome, while interactions between the enzyme and a different partner, protein B, might change the activity (Figure 1). Many non-enzymes also control access of substrates to the core enzyme, shaping its selectivity. For example, protein A might bind a specific substrate for the enzyme and thereby accelerate turnover by increasing its local availability. The assembly of multi-protein complexes is often mediated by a combination of strong and weak interactions between the individual protein components. Weak interactions are used to provide facile exchange of components (Perkins et al., 2010). Expanding beyond these immediate binding partners, multi-protein complexes often serve as “hubs” in a larger protein–protein interaction (PPI) network (Vidal et al., 2011). These ancillary interactions link the core and its partners to the broader cellular systems through a physical web of PPIs. It is becoming clear that chemical perturbations of a single node within the PPI network can have implications far beyond the immediate neighborhood (Conn et al., 2009).

Multi-protein complexes play critical roles in nearly all normal cellular functions, including gene expression, cell division, protein homeostasis, and signaling (Chari and Fischer, 2010; Good et al., 2011). Conversely, dysfunction in the assembly, localization, or dynamics of multi-protein complexes is associated with many diseases, including cancer, autoimmune disorders, and neurodegeneration. In some diseases, a complex may be aberrantly active; for example, in acute leukemia the *MLL* gene is translocated, resulting in fusion proteins between an N-terminal fragment of MLL and over 50 different target proteins (Liu et al., 2009). In other diseases, the function of a multi-protein complex may be disrupted, such as occurs in some p53 mutations (Muller and Vousden, 2013). While targeting the enzymatic components of multi-protein complexes has traditionally been the norm in drug discovery and in chemical biology, it is increasingly appreciated that PPIs could offer several advantages as targets (Hopkins and Groom, 2002; Wells and McClendon, 2007; Gordo and Giralt, 2009). For instance, this approach might allow disruption of some aspects of signaling cascades without completely shutting them down (Arkin and Whitty, 2009). Also, PPI interfaces tend to be more unique and varied than enzyme active sites, thus offering the possibility of greater selectivity (Bolanos-Garcia et al., 2012). As such, there is significant biological and therapeutic interest in developing chemical modulators of PPIs (Arkin and Whitty, 2009). Here, modulator is a term used to include both compounds that promote PPIs and those that inhibit PPIs. Such tools, provided they meet established criteria for chemical probes (Frye, 2010), are highly useful in revealing how specific PPIs are involved in normal function and pathobiology, as well as serving as starting points for therapies (Arkin et al., 2014).

**Figure 2:**
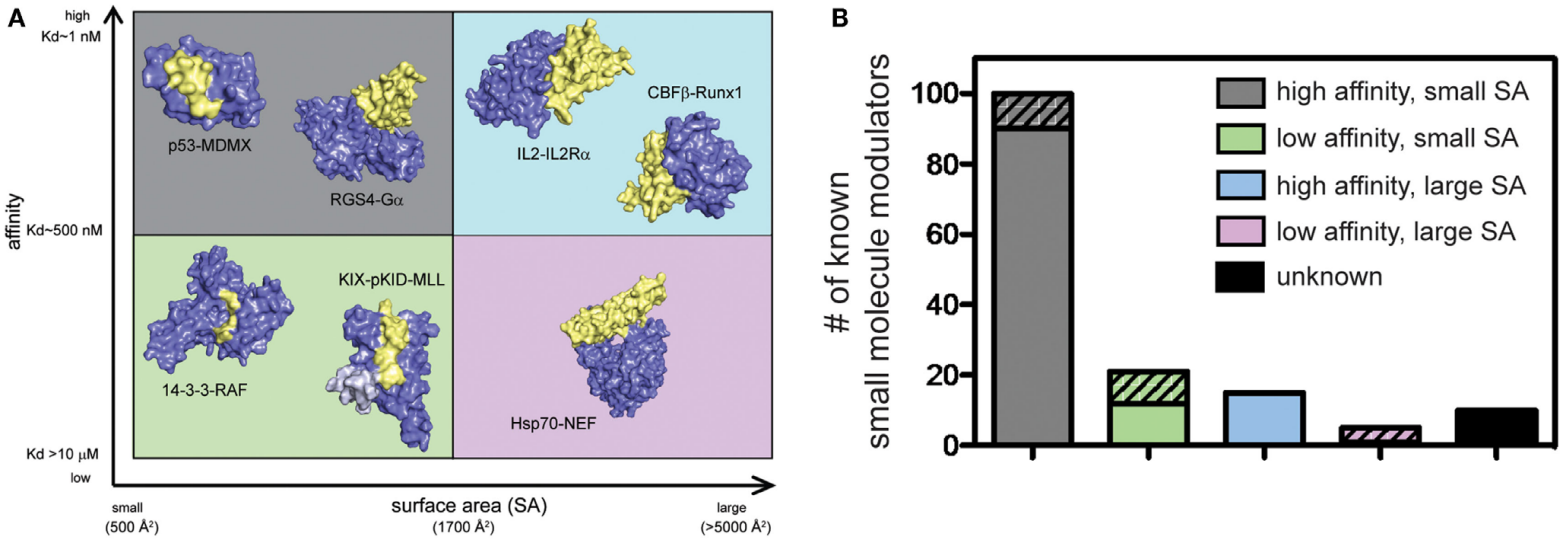
**Some protein–protein interactions may be more amenable to inhibition than others**. **(A)** PPIs categorized according to the apparent affinity of the interaction and the surface area (SA) buried by assembly. Application of arbitrary criteria for affinity (greater or less than 1 μM) and buried SA (greater or less than 1800 Å^2^) generates four quadrants. Examples of each class are shown. High affinity, small SA: p53−MDM2 (3DAC), RGS4−Gα_o_ (1AGR); high affinity, large SA: IL-2−IL-2Rα (1Z92), CBFβ−Runx1 (1E50); low affinity, small SA: GACKIX−pKID−MLL (2LXT), 14-3-3−RAF (3IQJ); low affinity, large SA: Hsp70−BAG1 (1HX1). **(B)** Known inhibitors of PPIs were collected from 2P2IDB and TIMBAL databases and plotted according to the affinity [reported in PDBbind (Wang et al., 2004)] and buried SA [calculated by InterProSurf (Negi et al., 2007)] of the target PPI. Inhibitors published since a similar analysis in 2012 (Thompson et al., 2012a) are represented by hatched bars to highlight the most recent development and trends. Small molecules targeting PPIs with high affinity and small buried SA represent 66% of all known inhibitors, although 56% of newly identified inhibitors target more difficult classes of PPIs.

PPIs have typically been challenging to disrupt with small molecules and, until relatively recently, these contacts have been classified as undruggable. It has become better appreciated that some PPIs may be more amenable to inhibition than others. For example, PPIs with relatively weak affinity and large surface areas (SAs) tend to be more challenging, while PPIs that rely on a few, closely spaced amino acids to bind with high affinity are relatively easier to inhibit (Overington et al., 2006; Wells and McClendon, 2007; Meireles and Mustata, 2011; Jubb et al., 2012). This point is demonstrated by the fact that of all known PPI modulators, the majority (>60%) target PPIs with affinity better than 1 μM and total buried SA less than 1800 Å^2^ (Figure 2). Another key observation is that, across many systems, orthosteric competitors are ideal for inhibiting the types of PPIs that are characterized by low SAs and tight affinity. For other types of PPIs, allosteric inhibitors tend to be more successful because they can exploit distal pockets that might have more favorable binding properties. Indeed, it is often the lack of defined binding pockets that makes it challenging to target small molecules to the PPIs that involve large, complex surfaces. In such cases, the free energy of binding is typically a summation of many low-affinity contacts, making it hard to design an effective competitor with low molecular weight (Smith and Gestwicki, 2012).

Another major theme of this review is that two (or more) chemical inhibitors that act on the same target can produce different cellular responses because of the way that they alter local PPI networks. Extensive work on allosteric modulation of G-protein coupled receptors (GPCRs) has provided a blueprint for how this might be accomplished in other systems (Keov et al., 2011). GPCRs signal through a series of well-characterized downstream PPIs (Luttrell, 2008). Allosteric and biased compounds offer an opportunity to have greater control over signaling by “fine-tuning” the response, illustrating the nuanced ways in which small molecules modulate the output of these systems (Christopoulos, 2014). A key observation from these examples is that binding of distinct ligands, even to the same site, can produce dramatically different effects on downstream signaling (Galandrin and Bouvier, 2006). For example, propranolol binds the β2 adrenergic receptor, although it is an inverse agonist for adenylyl cyclase signaling and an agonist for extracellular signal-regulated kinase activity. The field of kinase inhibitors may also provide another illustrative example. Kinase inhibitors fall into four general categories, the two most important of which are: type I and type II. Type I compounds bind directly to the ATP binding site in the kinase domain. Type II inhibitors, on the other hand, interact preferentially with the closed or inactive conformation via binding to an allosteric site (Liu and Gray, 2006). While both Type I and Type II inhibitors block enzymatic activity, they appear to have distinct effects on downstream PPIs between the kinases and their effectors. Why is this categorization important? For the sake of this review, type I and type II inhibitors might be expected to have different effects on the interactions between the kinase and its downstream effectors, such as 14-3-3 proteins, which link it to broader PPI networks. Thus, the effects of the inhibitors on cells might reflect both the inhibition of the kinase itself, but also the changes in PPIs.

In this review, we discuss several strategies for targeting PPIs with small molecules, with a focus on small molecules that take advantage of allosteric networks within multi-protein complexes. In the first section, we describe lessons learned from natural PPI modulators. We also review examples of synthetic molecules that have been discovered by serendipity to control PPIs through interesting mechanisms. In the second section, we survey a number of promising high-throughput screening (HTS) approaches that are geared toward the purposeful discovery of PPI modulators with similar mechanisms. Finally, in the third section, we speculate on which new methods and ideas might be needed to take full advantage of future opportunities. The over-arching theme is that small molecules have both proximal (or direct) effects on PPIs (e.g., they block or favor specific PPIs), while also having less well-appreciated effects on downstream interactions within PPI networks.

## Lessons Learned: Natural and Synthetic Examples of Allosteric Regulation of Protein Complexes

Allostery, in which binding of a ligand at one site affects protein conformation at a distant site, enables small molecules to produce dramatic effects on protein structure and function, even at a distance (Lindsley and Rutter, 2006; Weinkam et al., 2012). Classic work in this area was performed on the hemoglobin system, revealing that action at one binding site can propagate conformational changes that impact other sites more than 25 Å away (Monod et al., 1965). This theme has been observed countless times in biology, with allosteric control observed for both small molecule- and protein-mediated interactions. To name just one example, the activity of the histone deacetylase enzyme HDAC3 requires recruitment to a co-repressor SMRT complex (Li et al., 2000; Guenther et al., 2001). However, when expressed in bacteria, recombinant HDAC3 and SMRT do not interact, leading to speculation that an assembly factor was missing (Guenther et al., 2002). It was subsequently found that the deacetylase activation domain (DAD) of SMRT undergoes substantial structural rearrangement upon binding to HDAC3 and that an inositol tetraphosphate molecule was essential for this transition (Watson et al., 2012). The inositol molecule stabilizes the HDAC3–SMRT complex through conformational transitions involving both protein–protein and protein–small molecule contacts. What can be learned from these natural examples? In the HDAC3–SMRT case, a cascade of conformational changes occurs following the small molecule interaction. Thus, the small molecule needs to not only bind its target protein, but also alter the conformer of the target in the right way to enable subsequent binding to the downstream partner(s). In a broader sense, it seems possible that whenever a protein is bound by a small molecule, a specific subset of conformers is captured and those conformers might have important implications for what happens next.

One illustrative example of these concepts is the case of the retinoic acid receptor (RAR). In this system, gene expression is repressed when RAR is bound to a co-repressor, while gene expression is activated when RAR recruits a co-activator (Gronemeyer et al., 2004). The key structural feature is a switch between an extended β-sheet and α-helix in RAR, which occurs in a region that is important for binding to both co-repressors and co-activators (le Maire et al., 2010). Chemical agonists of RAR promote co-activator binding by stabilizing the correct, permissive conformation (Perissi and Rosenfeld, 2005), while inverse agonists convert the α-helix to an extended β-strand, promoting binding to co-repressors. Finally, neutral antagonists stabilize a conformer in which neither co-activators nor co-repressors are bound (Germain et al., 2009). Thus, depending on the chemical cue that is encountered (e.g., agonist, neutral antagonist, etc.), there are dramatic and important changes in PPIs that dictate downstream signals (le Maire et al., 2010). Such systems can be considered pharmacological “switches,” in which the local PPI network is re-wired by the small molecule.

Another useful example is the scaffolding protein family 14-3-3. These versatile adaptor proteins bind to hundreds of individual partners through a conserved amphipathic binding groove (Yaffe et al., 1997; Hermeking and Benzinger, 2006). 14-3-3 proteins are able to adopt many distinct conformations that allow them to interact with different binding partners (Johnson et al., 2010). Inhibitors exploit this property. For example, the natural product fusicoccin A promotes 14-3-3 complex assembly with some partners (Würtele et al., 2003; Ottmann et al., 2007), while the pyridoxal-phosphate derivative FOBISIN101 inhibits interactions with specific partners (Zhao et al., 2011). In these cases, the compound produces a specific cellular effect because it traps a conformation of the 14-3-3 protein and alters its PPI interfaces (Milroy et al., 2013).

Other examples drive home this idea, such as the case in which small molecules have been found to tune the activity of the important molecular chaperone, Hsp70. Dihydropyrimidines were identified that bind at the interface between Hsp70 and its PPI partner, Hsp40. The dihydropyrimidine remodels the PPI surface, such that some analogs strengthen the Hsp70–Hsp40 complex, while others inhibit it (Wisén et al., 2010). Similar concepts have been proposed for the transcriptional co-activator proteins, including the master co-activator CBP and components of the Mediator complex, in which allosterically coupled binding interfaces mediate interactions with transcriptional activators (Novatchkova and Eisenhaber, 2004; De Guzman et al., 2006; Vojnic et al., 2011). The theme in these systems is that the small molecule does not just alter enzyme activity – it impacts the way in which the protein partners recognize the target. Thus, we suggest that one of the most important features of a small molecule is how it traps a specific protein conformer. The ultimate biological output of a small molecule will be a product of the changes in both enzyme activity and its effects on PPIs networks.

### Inhibition of protein–protein interactions

**Figure 3:**
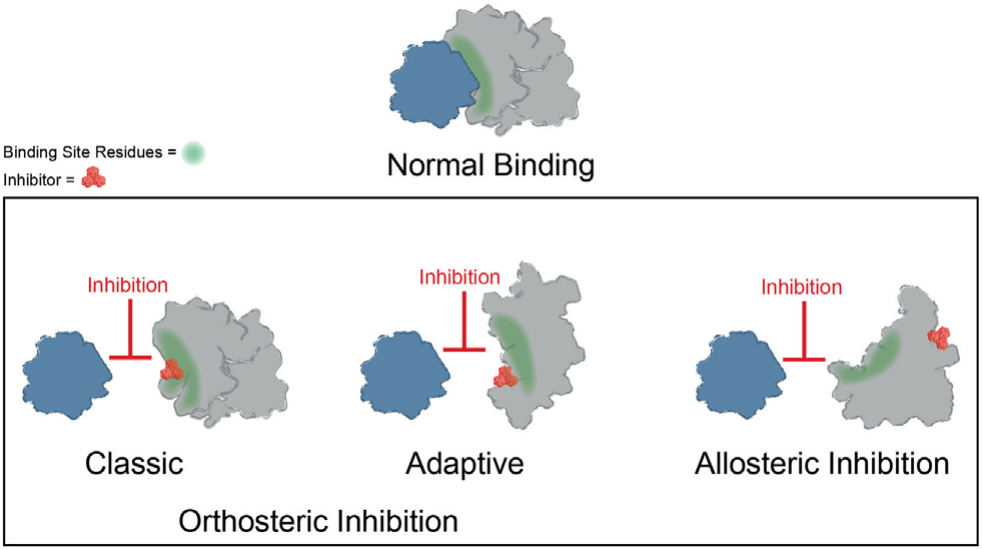
**Mechanisms of small molecule inhibition of protein–protein interactions**. Several different binding modes for small molecule inhibitors of PPIs are shown. Orthosteric inhibitor directly competes with one partner for binding. Orthosteric inhibitor taking advantage of an adaptive protein interface stabilizes a protein conformer such that the interaction surface is no longer amenable to binding. An allosteric inhibitor binds a site distal from the PPI interface, resulting in structural rearrangement in the target protein.

Significant progress has been made toward identifying orthosteric inhibitors of PPIs over the past 15–20 years (Wells and McClendon, 2007; Meireles and Mustata, 2011; Jubb et al., 2012; Arkin et al., 2014). Classic success stories include inhibitors of p53–MDM2 (Vassilev et al., 2004) and inhibitors of BCL2/BCL-X_L_ (Oltersdorf et al., 2005) and IAPs (Cai et al., 2011). In these cases, a molecule binds at the surface and directly prevents the most important “hotspot” side chains from interacting (Figure 3). In addition, the search for orthosteric inhibitors has revealed important features of the dynamics of PPI surfaces. For example, Tilley and coworkers reported the discovery of a small molecule that inhibits binding between the cytokine IL-2 and the IL-2 α receptor (IL-2Rα) (Tilley et al., 1997). Subsequent structural analysis suggested that this compound binds to IL-2 in a region that is critical for productive binding to IL-2Rα. Importantly, the unliganded IL-2Rα binding interface on IL-2 is dynamic and samples many distinct conformations, and binding of the small molecule restricts the total number of conformations sampled by IL-2, effectively “trapping” the protein in a conformation distinct from either the apo or IL-2Rα bound structures. Furthermore, the adaptive protein interface was more amenable to inhibitor discovery via disulfide tethering than an IL-2 subsite that is more conformationally restricted (Arkin et al., 2003). Many protein–protein interfaces are similarly adaptive, and while thought to be relatively flat and featureless, such binding interfaces can nonetheless sample conformations that allow for the formation of a small molecule binding pocket (Figure 3) (Brown and Hajduk, 2006; Wilson and Arkin, 2013). Why is it worth considering classic and adaptive orthosteric inhibitors as different classes? While both types ultimately inhibit the PPI by occluding the site of interaction, medicinal chemistry efforts to optimize them will depend on their mechanism. For example, classic orthosteric inhibitors do not induce substantial conformational rearrangement of the binding site, and therefore the apo structure of the protein can be used to guide the synthesis of new analogs. On the other hand, adaptive inhibitors require a conformation of their protein target that is distinct from the apo structure. Thus, structure-guided medicinal chemistry campaigns must be undertaken with this in mind.

Binding of small molecules at allosteric sites can also produce robust inhibition of PPIs (Figure 3). The interaction between Runx1 and CBFβ mediates formation of the heterodimeric transcription factor CBF (Speck et al., 1999). In some cases of acute myeloid leukemia, CBFβ is fused to the smooth muscle myosin protein, favoring formation of the CBFβ-Runx1 complex and resulting in dysfunction in CBF transcription (Liu et al., 1993; Castilla et al., 1996; Lukasik et al., 2002). Thus, inhibition of CBF heterodimer formation represents an attractive therapeutic strategy. Unfortunately, this interface is relatively large and featureless, characteristic of the difficult PPIs described previously. While attempts to discover orthosteric inhibitors of CBFβ-Runx1 binding have been unsuccessful, a combination of computational and NMR screens identified a class of 2-aminothiazoles that bind to an allosteric site on CBFβ distinct from the Runx1 binding interface and block CBFβ-Runx1 complex formation *in vitro* and in HEK293 cells (Gorczynski et al., 2007). Importantly, NMR chemical shift perturbations revealed that compound binding at the allosteric site produces changes in CBFβ conformation and/or dynamics at the heterodimerization site. Similarly, irreversible binding of a small molecule at an allosteric site on the regulator of G-protein signaling protein 4 (RGS4) produces more robust inhibition of binding to Gα_o_ than covalent modification within the binding interface (Roman et al., 2010). Temperature-accelerated molecular dynamics and NMR spectroscopy revealed how small molecule binding at an allosteric site on RGS4 is transmitted to the G-protein binding site in order to destabilize the PPI and block its GTPase accelerating activity (Vashisth et al., 2013).

Together, these studies suggest that allostery is a powerful approach for PPI inhibition and that it is particularly advantageous in circumventing the difficulties associated with challenging PPI interfaces, namely those with weak affinity and/or large SAs. This idea is illustrated in Table 1, in which the chemical structure of a subset of PPI inhibitors is listed next to the method used for their discovery, their mechanism of inhibition, and the class of the targeted PPI. This summary emphasizes the idea that allostery is a common property exploited by inhibitors of difficult targets and that certain discovery methods appear to be geared toward finding such molecules (as discussed below).

**Table 1:** **Summary of protein–protein interaction inhibitors**.

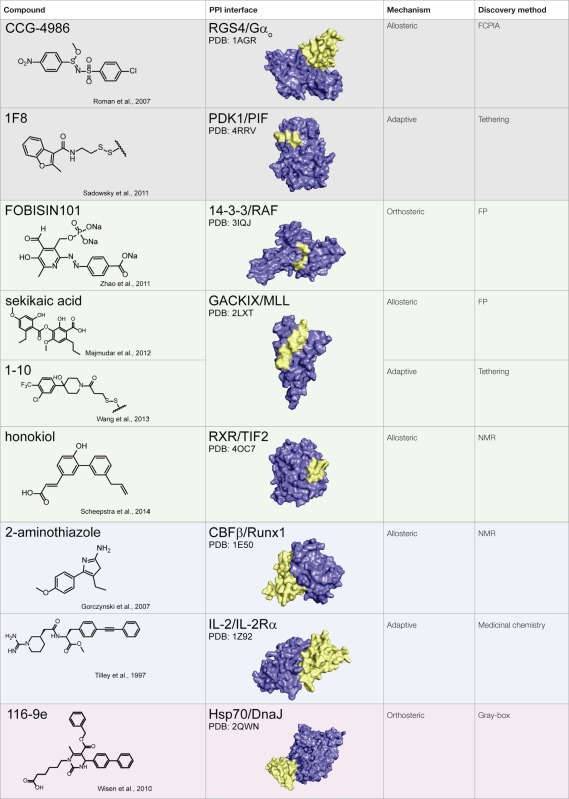

*High affinity, small SA (gray); low affinity, small SA (green); high affinity, large SA (blue); low affinity, large SA (purple)*.

### Promoting protein complexes

Small molecules can also be used to stabilize (rather than inhibit) PPIs, as described for the HDAC3–SMRT complex (Watson et al., 2012). In some cases, such potentiation can be therapeutically beneficial. Natural PPI stabilizers have been identified that nicely illustrate this idea (Giordanetto et al., 2014). The immunosuppressant cyclosporin A acts as a “molecular glue” between the peptidyl-prolyl *cis-trans* isomerase cyclophilin A and the protein phosphatase calcineurin (Huai et al., 2002). Similarly, FK506 stabilizes the interaction between the peptidyl-prolyl isomerase FKBP and calcineurin (Griffith et al., 1995). Another immunosuppressant, rapamycin, uses a similar approach to inhibit mTOR kinase (Brown et al., 1994). An example critical in plant development is the hormone auxin, which binds to TIR1 F-box proteins and stabilizes their interactions with Aux/IAA transcriptional repressor proteins (Delker et al., 2008). Finally, acyl-homoserine lactone (AHL) molecules are used by photobacteria in quorum sensing-mediated bioluminescence. AHL binding facilitates dimerization of LuxR-type transcription factors, increasing their DNA binding capacity and expression of target genes (Geske et al., 2008; Churchill and Chen, 2011). Importantly, in all cases, ternary complex formation is essential for function; that is, in the absence of small molecule, protein complex formation is negligible.

A number of synthetic stabilizers of PPIs have also been identified in recent years. For example, the murine double minute proteins MDM2 and MDMX are often overexpressed in cancer and negatively regulate p53-dependent gene expression (Marine et al., 2006; Toledo and Wahl, 2006). The tumor suppressor p53 controls pro-apoptotic and growth suppressing genes, and thus activation of p53-dependent transcription could have utility in cancer therapy (Wade and Wahl, 2009). However, inhibition of either the p53–MDM2 or p53–MDMX interaction alone was not sufficient to fully restore p53 signaling, and simultaneous inhibition of both PPIs is necessary for full activity (Tovar et al., 2006; Brown et al., 2009). Unfortunately, structural differences in the p53 binding sites of MDM2 and MDMX have hindered development of dual antagonists (Vassilev, 2005). Graves and coworkers at Roche Research Center instead identified a class of small molecules that inhibit both p53–MDM2 and p53–MDMX binding by inducing MDM2–MDMX protein dimerization, occluding the p53 binding site (Graves et al., 2012). While most reported examples of PPI stabilizers bind directly to the protein–protein interface, establishing contacts with both binding partners (those discussed in this review are summarized in Table 2) (Giordanetto et al., 2014), it is reasonable to speculate that small molecules might also be identified to stabilize specific PPIs through allosteric regulation, in which compound binding at a distal site would modulate the protein interface such that binding affinity is increased.

**Table 2:** **Summary of protein–protein interaction stabilizers**.

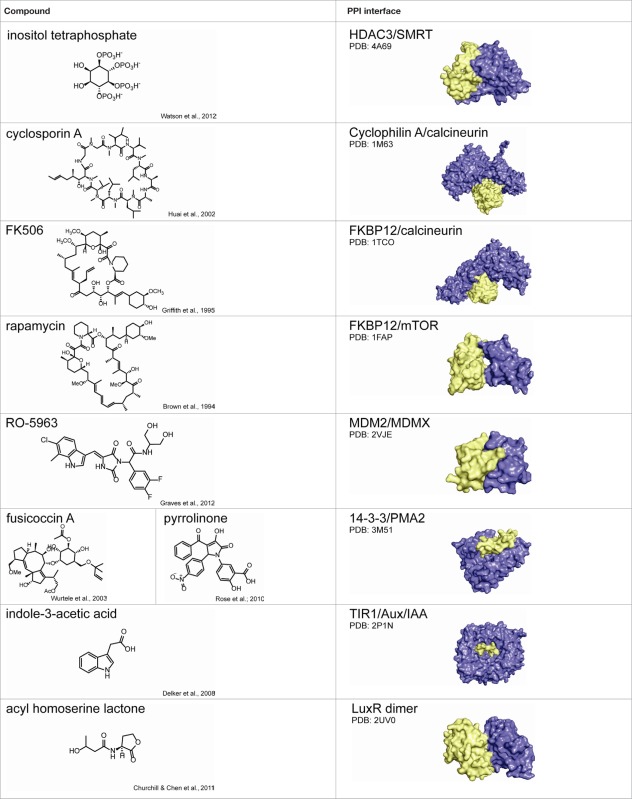

## Methods for Finding Modulators of Protein–Protein Interactions

Recent reviews have discussed the specific need for new methods in finding modulators of PPIs (Meireles and Mustata, 2011; Higueruelo et al., 2013). One identified challenge is that many traditional HTS methods rely on the measurement of direct binding between two protein partners. Such methods may not be suitable for finding potent inhibitors of some categories of PPIs, such as weaker ones. Another challenge is that very few methods are available that provide insight into the effects of a small molecule on broader PPI networks. This is particularly important for PPI inhibitor campaigns because screens must be specifically geared toward the discovery of molecules with the most suitable features, such as disrupting a subset of PPIs or favoring others (Kenakin, 2010). In other words, you get what you screen for.

**Figure 4:**
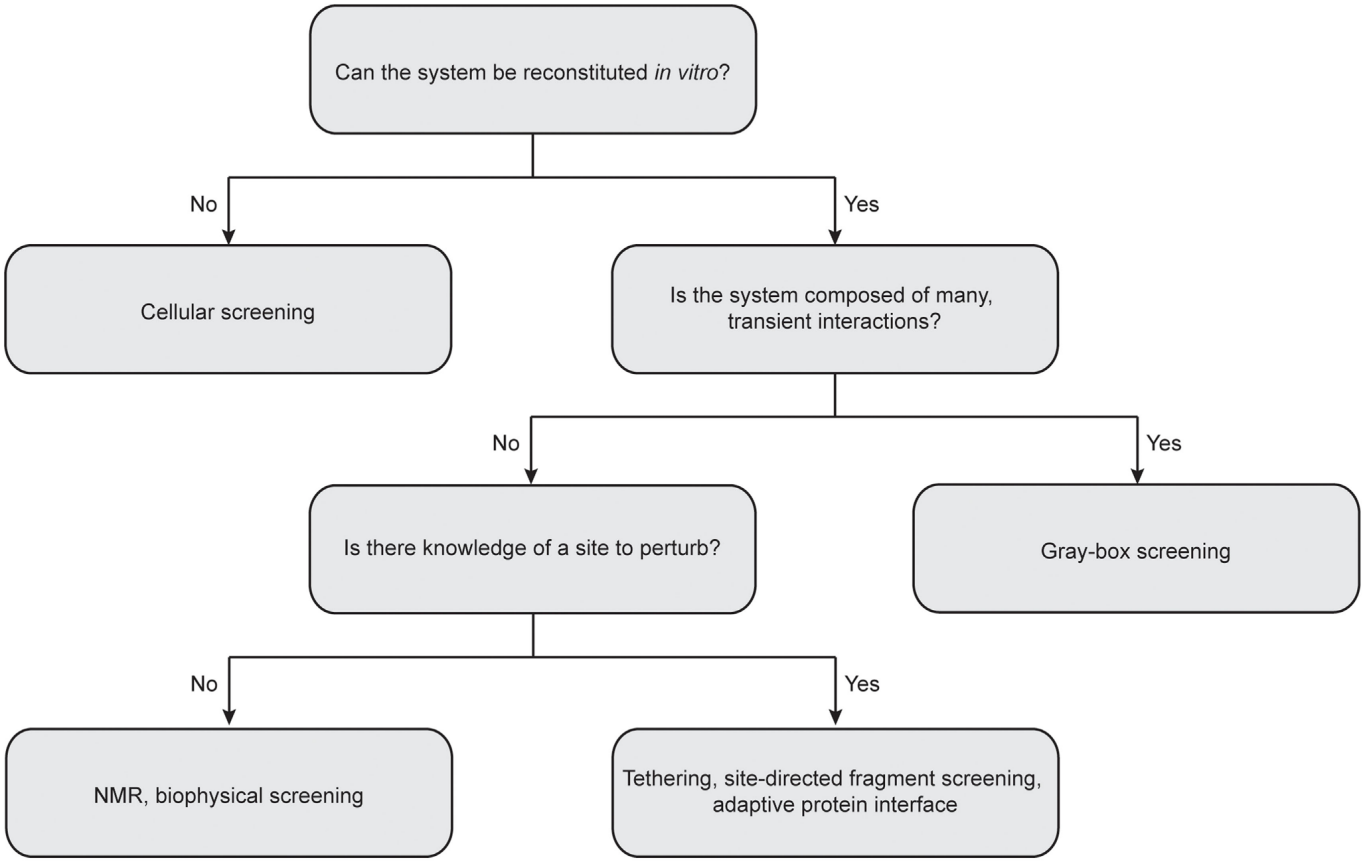
**Considerations for choosing the best approach for protein–protein interaction inhibitor discovery**. Central questions to consider include: Can the multi-protein complex be reconstituted *in vitro* with recombinant proteins? How stable is the interactions between individual components? Is there any precedent for sites to perturb? It is important to note that the “rules” for matching methods to targets are still in their infancy. Often, the best solution is to advance multiple platforms until the “best” solution is identified.

Due to the inherent complexity of multi-protein systems, it is difficult to provide a “one-size fits all” approach for future work, as a given HTS campaign must necessarily be geared for the complex of interest (Figure 4). For instance, many biophysical methods demand little advance knowledge of the complexities of a given system and require only purified recombinant protein. In addition, these approaches can quickly provide valuable information on protein and ligand-binding sites, allosteric networks, and druggable interfaces. On the other hand, cellular screening methods are well suited for multi-protein complexes composed of transient interactions that cannot be reconstituted *in vitro*. In this section, we highlight several successful approaches to discovering PPI modulators and comment on needs for the future.

### Biophysical methods

Some PPIs are difficult to directly measure, although assays based on fluorescence polarization (FP), surface plasmon resonance (SPR), fluorescence energy transfer (FRET), bioluminescence energy transfer (BRET), differential scanning fluorimetry (DSF), hydrogen-deuterium exchange, AlphaLisa, and NMR spectroscopy have been developed and each has its strengths (Holdgate et al., 2010; Makley and Gestwicki, 2012). FP is typically effective when the PPI involves a small SA (Nikolovska-Coleska et al., 2004); however, many PPIs occur over large, flat surfaces that lack such a discrete binding site. In these instances, a flow cytometry-based protein interaction assay (FCPIA) has proven to be a powerful, versatile alternative for PPI inhibitor discovery. In this method, one partner is biotinylated and attached to avidin beads, while the other partner is labeled with a fluorophore. A flow cytometer is used to measure bead-associated fluorescence, providing a quantitative measurement of protein binding (Sarvazyan et al., 1998; Sklar et al., 2002; Simons et al., 2003). FCPIA was used to discover inhibitors of the high-affinity interaction between a regulator of G-protein signaling protein RGS4 and Gα_o_ (K_D_ ~ 4 nM) (Roman et al., 2007). Importantly, RGS4 accelerates GTPase activity of Gα_o_, and inhibitors of the PPI also block GTPase stimulation. FCPIA has also been used to identify inhibitors of weaker PPIs, including that between Hsp70 and the BAG family of nucleotide exchange factors (K_D_ ~ 1 μM) (Rauch et al., 2013; Rauch and Gestwicki, 2014).

Another technique used to study protein complexes is capillary electrophoresis (CE). This method allows for separation of complexes from their individual components based on their size and charge. Labeling one or more of the individual protein partners enables sensitive detection of bound:free ratios (Schultz and Kennedy, 1993; Chu et al., 1995). CE has been used to identify inhibitors of a number of macromolecular complexes, including those between SH2 domains and short phosphorylated peptides (Yang et al., 2007) and Hsp70–BAG (Rauch et al., 2013). One advantage of CE is that it allows for easy detection of aggregators, a common problem in PPI inhibitor screens. Finally, both CE and FCPIA are compatible with multi-color fluorescent labeling, which facilitates the ready detection of ternary and higher order complexes in 384-well plate format.

NMR spectroscopy-based strategies are particularly useful for the detection of ligand binding to protein interfaces, even that those that involve modest affinity (e.g., high micromolar or millimolar dissociation constants) (Wendt et al., 2007). Furthermore, these experiments often illuminate the ligand-binding modes by chemical shift perturbations. This approach has been particularly powerful for fragment-based screens. Protein-observed NMR screening requires homogenous ^15^N or ^13^C isotopically labeled protein that has high solubility and stability even at high concentrations (Renaud and Delsuc, 2009). Isotopic labeling can be costly, and data acquisition for NMR spectra is often time-consuming. One alternative is ligand-based NMR screening and another is protein-observed fluorine NMR spectroscopy (PrOF NMR), an attractive approach with high sensitivity and rapid data collection. The ^19^F nucleus is highly responsive in NMR experiments and accounts for 100% of naturally occurring fluorine, eliminating the need for isotopic labeling (Kitevski-LeBlanc and Prosser, 2012). Native tyrosine residues are replaced with singly labeled fluorinated tyrosine, allowing for sensitive detection of protein conformation in simplified 1D-NMR spectra. This approach is particularly useful in the discovery of inhibitors of PPIs due to the enrichment of aromatic amino acids at PPI interfaces and has been used to characterize ligand binding to the transcriptional co-activation domain CBP/p300 GACKIX (Pomerantz et al., 2012) and bromodomains in BRD4, BrdT, and BPTF (Mishra et al., 2014). In addition, genetic incorporation of trifluoromethyl-phenylalanine was used to probe protein–ligand interactions within the thioesterase domain of fatty acid synthase (Cellitti et al., 2008). Recently, a full PrOF NMR fragment screen was used to discover several new small molecule ligands for distinct binding sites on CBP/p300 GACKIX, confirming the druggability of this dynamic protein interface (Gee et al., 2015).

Another emerging approach to get around the typical size limits of NMR spectroscopy is to label methyl groups on amino acid side chains. Methyl groups frequently reside in the hydrophobic interior of proteins and are sensitive reporters of protein structure and dynamics (Janin et al., 1988). NOEs can be used to determine spatial proximity of methyl groups, reporting on tertiary and quaternary protein structure (Rosen et al., 1996; Gardner et al., 1997). Furthermore, methyl groups located at the end of side chains undergo slower relaxation kinetics, facilitating the acquisition of NMR spectra for large proteins with high sensitivity and resolution (Nicholson et al., 1992; Tugarinov and Kay, 2005). Researchers at Abbott Laboratories adapted this approach to screen a series of protein targets using selective ^13^C labeling of methyl groups in valine, leucine, and isoleucine side chains, demonstrating the utility of this strategy in HTS for target proteins up to 110 kDa in size (Hajduk et al., 2000).

### Site-directed fragment-based screening

Covalent disulfide trapping, or Tethering, can be used in HTS to facilitate discovery of PPI inhibitors. In this method, fragment molecules containing disulfide moieties covalently modify a natural or engineered cysteine. The relative strength of the interaction is typically measured by competition with DTT or similar thiol modification reagent, permitting the selection of fragments that have the best affinity for the site (Erlanson et al., 2000, 2004). Binding is typically explored by crystallography, SPR or mass spectrometry. Tethering is a particularly powerful technique because it is site-directed. Indeed, Tethering screens have demonstrated that adaptive, conformationally flexible regions of protein–protein interfaces are often more amenable to inhibitors (Buck and Wells, 2005; Buck et al., 2005; Scheer et al., 2006). Tethering was recently used to successfully define the role of an allosteric site in activation of the PDK1 kinase. Engineered cysteines lining the rim of the PDK1-interacting fragment (PIF) pocket on PDK1 were screened for disulfide capture against a library of fragment molecules (Sadowsky et al., 2011). Interestingly, tethered fragments at a single site resulted in both activation and inhibition of PDK1, and the allosteric effect was unrelated to binding potency. Rather, fragment binding at a single allosteric site elicited subtle changes in the active conformation of PDK1, leading to potentiation or inhibition of kinase activity. Another recent example identified inhibitors of a specific cysteine mutant of Ras (Ostrem et al., 2013).

Fragments identified from Tethering screens can be powerful chemical probes for dissecting allosteric networks in flexible proteins. For example, the GACKIX domain of the transcriptional co-activator protein CBP/p300 displays considerable structural plasticity (Thakur et al., 2014). A recent Tethering screen yielded a small molecule (1-10) that has a remarkable effect on the overall thermal stability of GACKIX and was used as an aid in crystallization in order to obtain the first X-ray crystal structure of GACKIX at 2 Å resolution (Wang et al., 2013). More recently, 1-10 was used in conjunction with kinetic and computational analyses to reveal the mechanism of allostery between two activator-binding sites in GACKIX. Binding of one ligand does not affect the association kinetics for the second partner, but rather decreases the rate of dissociation of the complex (Wang et al., 2014). These results suggest that cooperativity between the two activator-binding sites is achieved by increasing the energy barrier for dissociation, effectively stabilizing the GACKIX ternary complex (Law et al., 2014). In other words, inhibition of activator binding is mediated by favoring the unbound binary complex.

### Adaptive protein interfaces

One screening approach is to take advantage of the conformational plasticity of PPIs. Protein surfaces that mediate contacts with a variety of other proteins are typically flexible, allowing them to adapt to each partner. For example, the GACKIX domain of the transcriptional co-activator CBP/p300 is an important regulatory node of gene expression and mediates binding to diverse transcriptional activators via two allosterically coupled binding sites (De Guzman et al., 2006; Brüschweiler et al., 2009; Thakur et al., 2014). Majmudar and coworkers reasoned that small molecules could take advantage of this intrinsic motion to trap specific states (Majmudar et al., 2012). GACKIX was screened with the activator MLL bound in the deeper and more conformationally flexible binding site, and this approach identified two natural products, sekikaic acid and lobaric acid. These compounds bind to a dynamic site on CBP/p300 GACKIX and allosterically inhibit interactions at the distal binding site with good specificity. In this case, knowledge of the adaptive, local PPI network was used to establish a screening paradigm that favored discovery of the desired probe.

Another recent example focused on nuclear receptors and their co-activator proteins. The retinoid X receptor (RXR) contains a dynamic protein interface, termed activation function 2 (AF2) that mediates interactions with co-activator proteins (Wurtz et al., 1996; Huang et al., 2010). Sheepstra and coworkers used a combination of NMR spectroscopy and molecular docking to identify a natural product honokiol that targets both sides of the adaptive AF2 domain (Sheepstra et al., 2014). They used rational design to “split” the two functions of honokiol, generating a potent RXR agonist with one and an antagonist with the other. The first ligand inhibits the RXR-coactivator PPI by binding at an allosteric site, while the second ligand binds directly to the ligand-binding site on RXR to favor interactions with the co-activator. Like the example of the CBP/p300, this approach exploits the inherent conformational flexibility of the AF2 domain and demonstrates how binding of small molecules at the same protein interface can differentially modulate specific PPIs.

Another interesting example involves the adaptor protein 14-3-3, which was introduced earlier. Binding between 14-3-3 and the plant proton pump PMA2 was monitored by SPR, and a small compound library was screened for stabilizers of the interaction (Rose et al., 2010). This campaign identified small molecules that promote the PPI between 14-3-3 and PMA2 by binding to the flexible binding groove on 14-3-3 and making important contacts with both partners. Synthetic optimization of the pyrrolinone scaffold in one of these molecules resulted in the development of a derivative that further stabilized the 14-3-3−PMA2 interaction (Richter et al., 2012). This example is interesting because the screening method was designed such that the two partners were near their half-maximal concentrations, which likely facilitated the discovery of the “hits”. Most screening campaigns involve saturating concentrations of the two interacting partners; not surprisingly, inhibitors are more commonly observed under these conditions.

These successful HTS campaigns demonstrate how a conformationally dynamic protein can be targeted with small molecules to specifically modulate the assembly and disassembly of multi-protein complexes. A key design feature of these screens is that structural knowledge of the system was used to guide the screen and favor identification of compounds with the desired mechanism.

### “Gray-box” screening

In many cases, weak binding affinity between a protein and its binding partner can complicate the design of an effective screening assay. In these cases, it can be useful to screen the functional output of the interaction, rather than the physical interaction itself. A method referred to as “gray-box” screening (Wong, 2011) was developed specifically for this purpose. The name of this method comes from the term “black box” screens, which is applied to phenotypic assays. While screens in whole cells or organisms are powerful, it is often difficult to find the target. Likewise, biochemical screens against purified proteins, such as kinases or proteases, are clean, but they often ignore the impact of PPIs and non-enzyme partners. Gray-box screens are designed to include not just the enzyme, but also its binding partners. In the first example of this approach, the ATPase, Hsp70, was mixed with its co-chaperones, including Hsp40. These co-chaperones act as catalysts of Hsp70’s ATPase activity (Harrison et al., 1997; Wittung-Stafshede et al., 2003; Ahmad et al., 2011), so the readout of the screen was largely a result of the PPIs and not the enzyme activity *per se*. Thus, the functional consequence of the interactions, measured as elevated turnover, can be used as a surrogate for binding (Chang et al., 2008; Miyata et al., 2010). This type of approach has identified many specific chemical modulators of PPIs between Hsp70 and co-chaperones, ATPase activity, and chaperone function (Wisén and Gestwicki, 2008; Wisén et al., 2010; Chang et al., 2011). Indeed, a screen against all possible binary and ternary complexes in the prokaryotic Hsp70 system identified inhibitors that were specific for given co-chaperones, each taking advantage of previously unexplored allosteric networks to inhibit Hsp70 activity (Cesa et al., 2013). Such diverse inhibitors are likely to be powerful tools and could enable the definition of Hsp70 PPI networks in a cellular environment. Gray-box screening has also been used to identify inhibitors of the interactions between Gα proteins and regulators of G-protein signaling (RGS) proteins, which act as GTPase activating proteins (GAPs). In this case, GTPase activity was used as a surrogate for RGS-Gα binding, and this screen identified several molecules capable of targeting the specific interactions between different RGS proteins and Gα_i_ (Monroy et al., 2013). In another example, the progesterone receptor (PR) was reconstituted with Hsp90 chaperone complexes, and compounds were screened for their ability to inhibit refolding of PR, a physiological substrate of Hsp90 (Patwardhan et al., 2015). Overall, gray-box screening is particularly well suited to finding inhibitors of challenging PPIs.

### Screening in cells

Some PPI networks cannot be readily replicated *in vitro*, demanding the use of cell-based screening platforms. However, even for a well-defined PPI, cell-based screens of the native network will likely yield modulators of up- and downstream interactions, required substantial deconvolution. To circumvent these challenges, enzyme fragment complementation can be used, allowing for a specific PPI to be screened in the cellular environment such that the functional output of the assay is dependent solely on the PPI of interest (Shekhawat and Ghosh, 2011). In this approach, protein fragments derived from enzymes such as luciferase, β-galactosidase, or dihydrofolate reductase are fused to putative interacting partners. The inactive fragments can reassemble into a functional complex upon interaction of the protein partners. Reconstituted enzymatic activity is used to quantify protein binding, even between transiently interacting partners.

The split luciferase assay can be used to detect PPIs in cells (Luker et al., 2004) and can be expanded to include a Cre-recombinase-mediated cassette exchange (Wong et al., 2005) allowing for inducible gene expression. The recombinase-enhanced bimolecular luciferase complementation platform (ReBiL) allows for the detection and analysis of even weak PPIs in living cells in real time. This platform enabled the detection of the transient PPI between the E2 ubiquitin-conjugating enzyme UBE2T and its partner E3 ubiquitin ligase FANCL (Li et al., 2014). This complex has a reported low micromolar dissociation constant and cocrystallization required the creation of a fusion protein between UBE2T and FANCL (Hodson et al., 2011, 2014). Unsurprisingly, this complex has previously eluded detection in living mammalian cells, although the ReBiL platform was able to readily detect the interaction. Furthermore, this assay was used to evaluate several reported small molecule and peptide antagonists of p53–MDM2 and p53–MDM4 interactions (Li et al., 2014). In particular, SAH peptides did not disrupt complex formation between p53 and either MDM2 or MDM4; rather, their previously reported cellular activity could be contributed to p53-independent cell membrane disruption. Importantly, ReBiL was readily adapted to 1536-well format, making it a powerful, high-throughput technique for the detection of even weak or transient protein complex formation in real time in living cells.

## Dissecting Protein Networks *In Vitro* and in Cells

**Figure 5:**
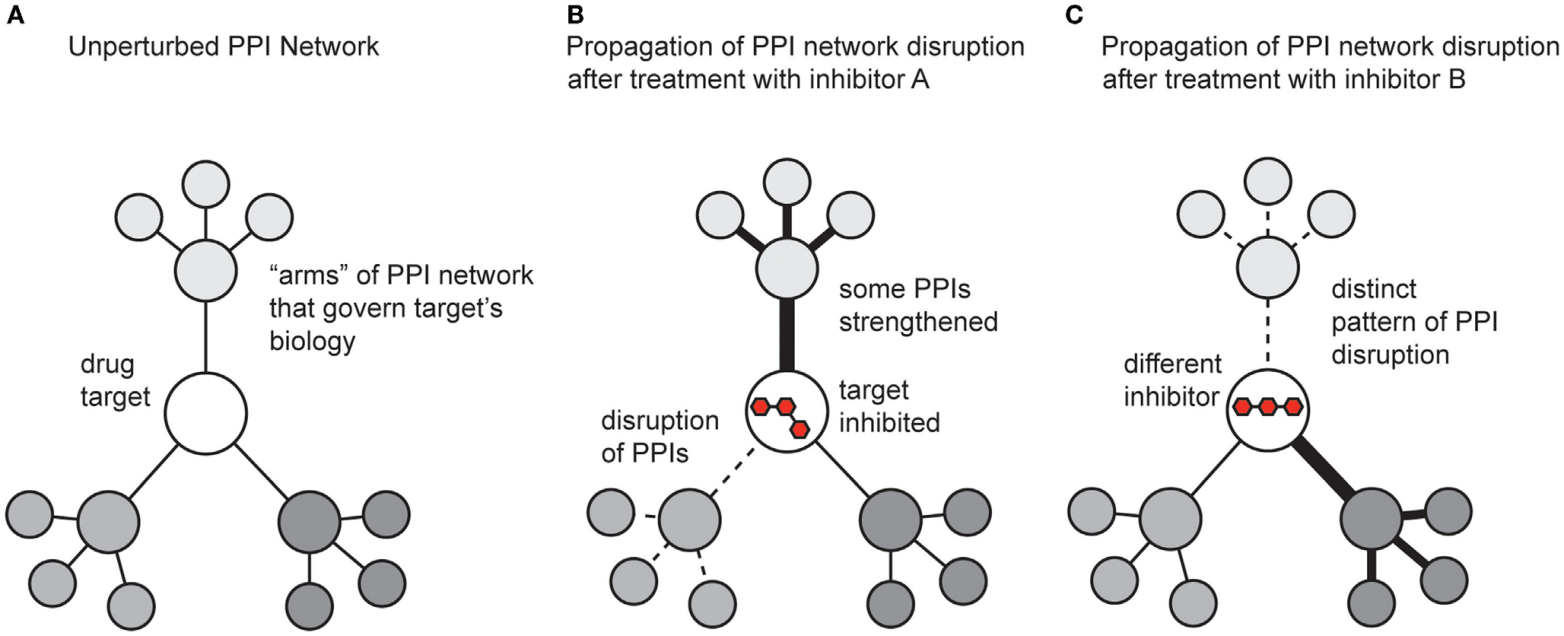
**Schematic representation of how small molecules might propagate changes in protein–protein interaction networks**. **(A)** A theoretical drug target interacts with multiple proteins, which connect it to the broader PPI network. **(B)** Treatment with an inhibitor might weaken some PPIs (dotted lines) and strengthen others (dark lines). The ultimate response to the inhibitor is manifested by both the direct effect on its target and the new state of the perturbed PPI network. **(C)** A different inhibitor, even acting on the same target, might generate a non-overlapping phenotype by trapping the target in a state that perturbs the network in distinct ways.

While the previous sections focused on methods for discovering modulators of PPIs, it is also critical to understand the impact of the molecules on broader PPI networks. A key tool here continues to be mass spectrometry. Many groups have developed methods for measuring and quantifying PPI networks in cells (Vidal, 2005). What has been less well explored is how small molecules affect these systems. This is somewhat surprising, and a more concerted effort to study how compounds change PPI networks will provide significant insight. Inhibiting a single protein target with a small molecule affects not only the direct interactions between the target and its partners, but also propagates changes throughput the entire protein network (Figure 5). In addition, the shape of the ligand and the accompanying conformer of the bound protein target dictate how changes are transmitted throughout the overall protein network, both in the extent of modulation and which “arms” of the network are affected. One might envision that some inhibitors might act on the same target, but produce different outcomes because of their unique pattern of affected PPIs (Figure 5).

Proteins and other macromolecules exist in an ensemble of conformational states, and binding to other macromolecules or ligands can have profound effects on their dynamics (Boehr et al., 2006; Henzler-Wildman et al., 2007). Each member of the structural ensemble has the potential to bind different partners or perform specific tasks (Tang et al., 2006). As discussed, many small molecules trap particular protein conformations and this state can propagate important downstream effects (Jubb et al., 2015). Thus, one powerful method is to explore time-dependent perturbations in protein structure (Henzler-Wildman and Kern, 2007; van dem Bedem and Fraser, 2015). For example, pioneering efforts in nanoflow electrospray ionization (nESI) have revolutionized the study of large protein complexes with its increased sensitivity and preservation of weak non-covalent interactions (Hopper et al., 2012). Because detection occurs in the gas phase, this technique effectively captures a “snapshot” of a binding equilibrium that exists in solution. Furthermore, multiple protein partners can be detected simultaneously within the context of the larger assembly without the need to isolate specific complexes (Hopper and Robinson, 2014). nESI is therefore particularly suited to the study of multi-protein complexes in real time. Recently, it has been used to quantify the assembly of complexes between the molecular chaperones Hsp90, Hsp70 and the co-chaperones FKBP52 and HOP (Ebong et al., 2011), as well as polydispersed oligomers of small heat shock proteins (Aquilina et al., 2005).

Ion mobility-mass spectrometry (IM-MS) is a technique that separates macromolecules in the gas phase, analogous to electrophoresis in solution. Measurement of ion migration, or drift time, can be used to generate information on the collisional cross section of a protein of interest, which can in turn be used to infer changes in the folded conformation of a protein upon ligand or macromolecule binding (Niu et al., 2013). The introduction of collision-induced unfolding measurements enable IM-MS to report on more subtle changes in protein tertiary structure induced by ligand binding (Hopper and Oldham, 2009; Hyung et al., 2009). This development has been useful for evaluating different binding modes of similar ligands, in particular the subtle differences in kinase structure upon binding to type I and type II inhibitors (Rabuck et al., 2013). This approach was sensitive enough to cluster several well-characterized type I and type II Abl kinase inhibitors based on their preferred protein structure and has the potential to be adapted to a larger screen of a chemical library for evaluation of ligand-induced changes in protein conformation.

### How can small molecules produce diverse cellular outcomes via hitting the same target?

It is important to consider that small molecule modulators of PPIs are often not simple, functional “on-off switches.” Rather, compounds can have sundry effects on function because the immediate PPIs are linked to the broader network. To illustrate this complexity, it is worth considering the example of Hsp70 inhibitors. Hsp70 is an important molecular chaperone that regulates protein quality control through a conserved mechanism of ATP hydrolysis and substrate binding. Many classes of Hsp70 inhibitors have been identified and each of these inhibits nucleotide turnover *in vitro* (Patury et al., 2009; Evans et al., 2010). Thus, one might assume that each of these compounds have similar downstream effects on Hsp70’s functions in cells. However, this assumption turns out to be incorrect because each category of inhibitor has a unique impact on Hsp70 PPIs. Efforts by multiple groups have provided Hsp70 “inhibitors” that either compete with ATP or inhibit interactions with specific classes of co-chaperones (Schlecht et al., 2013). In each case, the compound blocks steady-state ATPase activity *in vitro*, yet the cellular effects are not equivalent. For example, rhodacyanines that inhibit PPIs between Hsp70 and its nucleotide exchange factors (Li et al., 2015) lead to dramatic degradation of the polyglutamine expanded androgen receptor (PolyQ-AR) (Wang et al., 2012), a well-established Hsp70 client, while molecules that inhibit ATP binding (Miyata et al., 2012) lead to substantial increases in accumulation of polyQ-AR (Wang et al., 2010). Therefore, two classes of molecules with equivalent ability to inhibit ATPase activity have opposing effects on polyQ-AR levels, although the exact mechanisms that link these events to cellular outcome are not yet clear. Nonetheless, molecules targeting the same protein can produce distinct outcomes, likely due to differences in the way that the target, in this case Hsp70, engages with downstream partners. An increased understanding of how protein networks are linked to disease may eventually allow for a more rational approach to drug discovery and improved screening paradigms that more correctly predict outcomes. In many ways, these observations mirror what is observed in GPCR agonists/antagonists.

### Small molecule-induced changes in local and global protein networks

Emerging large-scale PPI maps have been instrumental in defining the protein interactome in mammalian cells (Rual et al., 2005; Parrish et al., 2006). Advances in quantitative mass spectrometry, including its combination with affinity purification (AP-MS), have allowed for dynamic profiling of PPIs at near physiological conditions (Gingras et al., 2007). In this technique, a protein of interest (bait) is expressed with an epitope tag, which is used to purify the bait protein from cell lysate along with its interacting protein partners (prey) (Dunham et al., 2012). Commonly used tags include the short FLAG tag or the tandem affinity purification (TAP) tag. The TAP approach requires two affinity tags separated by a protease cleavage site (Gavin et al., 2006). Purification of the bait and prey proteins from cell lysate occurs over two steps, and this strategy can decrease the identification of false positive proteins in subsequent analysis. Tandem mass spectrometry is then used for identification and quantitation of the isolated proteins. This powerful approach allows for rapid sequencing and identification of thousands of individual peptides, including characterization of post-translational modifications (Aebersold and Mann, 2003; Nesvizhskii, 2007). Furthermore, mass spectrometry is readily adapted to quantify protein abundance in the original sample, which can provide insight into the dynamics of protein complex assembly (and disassembly) in response to pharmacological manipulation (Ong et al., 2003). It is important to note that the lack of detection in an AP-MS experiment does not imply a lack of interaction between the bait protein and a prey protein of interest. The stringency of washing conditions can disrupt transient PPIs, which can complicate analysis (Gingras et al., 2007).

Despite these limitations, many groups have nonetheless successfully used AP-MS to identify and characterize protein complexes in living cells in response to pharmacological manipulation. In *Saccharomyces cerevisiae*, polyglutamine (polyQ) fragments form cytosolic aggregates, and this process is regulated by molecular chaperones (Rikhvanov et al., 2007). The dihydropyrimidine 115-7c promotes binding of Hsp70 to Hsp40 and polyQ, increasing polyQ solubility. AP-MS revealed changes in proteins bound to polyQ fragments as a function of polyQ length, aggregation time, and 115-7c treatment (Walter et al., 2011). In a similar study, Thompson and coworkers used AP-MS to study acute changes in the interaction network associated with turnover of microtubule-associated protein tau (Thompson et al., 2012b). Several proteins, including Hsp70 and Hsp90 had differential association with tau following treatment with an inducer of tau degradation. In both examples, MS was a powerful tool for dissecting the dynamic changes in multi-protein complexes in response to pharmacological disruption of PPIs.

A major hurdle to AP-MS is the difficulty in identifying partners with low affinity and/or low abundance. Several groups have developed creative solutions to this challenging problem, including crosslinking. Examples of synthetic crosslinkers include activated diesters linked by a cyclic quaternary diamine. The diamine is cleavable by collision-induced dissociation and facilitates identification of crosslinked peptides (Clifford-Nunn et al., 2011). Another crosslinking method is the use of genetically encoded photoactivatable amino acids, such as *p*-benzoyl-l-phenylalanine (Bpa), which are used to covalently and site-specifically capture PPIs in their native environment (Chin and Schultz, 2002; Hino et al., 2005). This approach has been successfully adapted to both stable, high-affinity PPIs as well as more transient, moderate- to low-affinity PPIs within the transcriptional machinery. Photoactivation of Bpa encoded in the transcriptional activator Gal4 captured its stable interaction with the suppressor protein Gal80 (Majmudar et al., 2009a). Interactions of transcriptional activators with co-activator proteins are typically much more transient (Fuxreiter et al., 2008). For example, the Swi/Snf chromatin-modifying complex is a proposed binding target for the viral activator VP16, although the specific interactions had evaded detection with traditional methods (Neely et al., 2002). Photo-crosslinking of Bpa confirmed that VP16 makes direct contacts with both Snf2 and Snf5 during transcription initiation (Krishnamurthy et al., 2011). Because this approach is site-directed, it can be combined with mass spectrometry to localize interaction “hot spots” for specific PPIs and to identify and characterize novel binding partners for a protein of interest (Majmudar et al., 2009b).

A recently reported proximity biotinylation approach, BioID is a complementary method for mapping specific PPIs within large multi-protein complexes. This strategy is particularly advantageous because it does not require that complexes be maintained across numerous purification steps and therefore has the potential to identify more transient PPIs compared to traditional AP-MS techniques (Roux et al., 2012). In this method, the bait protein is fused to a mutated prokaryotic biotin ligase BirA. This enzyme covalently links acceptor proteins with biotin via an activated intermediate (biotinoyl-5′-AMP). However, the mutant enzyme dissociates rapidly from the activated biotinoyl-5′-AMP, creating a “cloud” of activated biotin surrounding the bait protein (Kwon and Beckett, 2000). The activated biotin can then covalently modify exposed lysine residues on the prey proteins, which can include direct partners as well as neighboring proteins. Alternatively, specific prey proteins can be fused with an acceptor peptide for the biotin ligase (Fernández-Suárez et al., 2008). Rather than subsequent affinity purification with the tagged bait protein, prey proteins are enriched with streptavidin purification and identified by mass spectrometry. This approach has been successfully adapted to the *in vivo* characterization of a number of diverse, dynamic protein complexes, including the chromatin-associated mediator complex (Lambert et al., 2014), members of the nuclear lamina (Roux et al., 2012) and nuclear pore complexes (Kim et al., 2014), and components of the inner membrane complex in *Toxoplasma gondii*, among others (Chen et al., 2015). These complementary approaches, including AP-MS, protein crosslinking, and proximity biotinylation can be used in tandem to create a full picture of a protein complex in living cells, as each approach has the potential to identify novel interactions.

## Conclusion and Outlook

Multi-protein complexes are the “hubs” of the cellular PPI networks and attractive drug targets for a variety of diseases. We have illustrated in this review several “success stories” of small molecules that target PPIs and the development of new methodology for identifying new PPI modulators. Creative HTS strategies are beginning to expand the toolbox of available approaches, although there is no algorithm or “road map” for a successful screen. Rather, each campaign must be designed individually, taking into account the affinity of the interactions, the topology of the interaction surfaces, and the interplay between different components of the system. For instance, a phenotypic or gray-box screen has the best chance of success for finding small molecules that can perturb protein networks. However, these strategies require significant knowledge of the structure and function of individual PPIs within a greater protein complex, highlighting the importance of basic research in order to be able to ask the right questions.

## Conflict of Interest Statement

The authors declare that the research was conducted in the absence of any commercial or financial relationships that could be construed as a potential conflict of interest.
